# Bridging the gap in outpatient care: Can a daily patient‐reported outcome measure help?

**DOI:** 10.1002/cnr2.1421

**Published:** 2021-07-10

**Authors:** Andreas Meryk, Gabriele Kropshofer, Benjamin Hetzer, David Riedl, Jens Lehmann, Gerhard Rumpold, Alexandra Haid, Bernhard Holzner, Roman Crazzolara

**Affiliations:** ^1^ Department of Pediatrics Medical University of Innsbruck Innsbruck Austria; ^2^ Department of Psychiatry, Psychotherapy and Psychosomatics University Hospital of Medical Psychology, Medical University of Innsbruck Innsbruck Austria; ^3^ Department of Psychiatry, Psychotherapy and Psychosomatics University Hospital of Psychiatry II, Medical University of Innsbruck Innsbruck Austria

**Keywords:** childhood cancer, daily web‐based, ePROtect, patient‐reported outcome measure, symptoms

## Abstract

**Background:**

Childhood patients have high risks for developing debilitating somatic and mental health side‐effects as a consequence of the many different approaches employed in treating their cancer. Early recognition and close monitoring of clinical and psychological problems are essential in planning appropriate interventions and preventing further deterioration.

**Case:**

ePROtect was established as an easy‐to‐use application for daily self‐reporting of symptoms during cancer therapy. ePROtect includes six to eight questions pertaining to seven common symptoms: appetite loss, fatigue, nausea, pain, physical functioning, cognitive impairments and sleep quality. The case of a child diagnosed with Burkitt leukemia developing chemotherapy‐induced oral mucositis in home care is presented to show the therapeutic impact of early symptom detection with a daily web‐based tool.

**Conclusion:**

This case highlights how electronic patient‐reported outcome measures (PROM) can directly facilitate patient care in real time and might be incorporated in future clinical routine.

**Abbreviations**
CHES: computer‐based evaluation system
COVID‐19: coronavirus disease 2019
CRP: C‐reactive protein
ISPOR: International Society for Pharmacoeconomics and Outcomes Research
PedsQL: pediatric quality of life inventory
PROM: patient‐reported outcome measures

## 1 INTRODUCTION

In a matter of months, the coronavirus disease 2019 (COVID‐19) massively changed everyday life, prompting multiple responses to meet this unprecedented challenge.^1^ Children undergoing cancer treatment is particularly seriously affected. In addition to being afraid of treatment, they are also afraid of being infected with SARS‐CoV‐2 and on a daily basis are confronted with prevention measures, including restricted access to the healthcare system.^2^ In this scenario and long before the pandemic, it was recognized that the patient himself had no possibility to report symptoms and that child‐specific measures for capturing health outcomes should be urgently developed.^3^, ^4^, ^5^

With the aim of helping overcome obstacles in the routine measurement of patient‐reported symptoms in the treatment of childhood cancer, we developed a unique web‐based approach for daily child self‐report. For the purpose of illustration, a first anecdotal case is presented and future challenges in facilitating the success and sustainability of interventions by healthcare providers are discussed.

## 2 CASE

Daily self‐reporting was conducted via ePROtect (https://ches.tirol-kliniken.at/protect-portal), an interface based on the CHES software (computer‐based evaluation system) and established as an easy‐to‐use application for patients during cancer therapy.^6^ ePROtect includes six to eight questions adapted from the Pediatric Quality of Life Inventory (PedsQL) 3.0 Cancer Module pertaining to seven common symptoms: appetite loss, fatigue, nausea, pain, physical functioning, cognitive impairments, and sleep quality. The monitoring questionnaire assesses symptom burden in those domains on the previous day. The impairment was graded ranging from zero (not at all bothered) to four (severe impairment).

**FIGURE 1:**
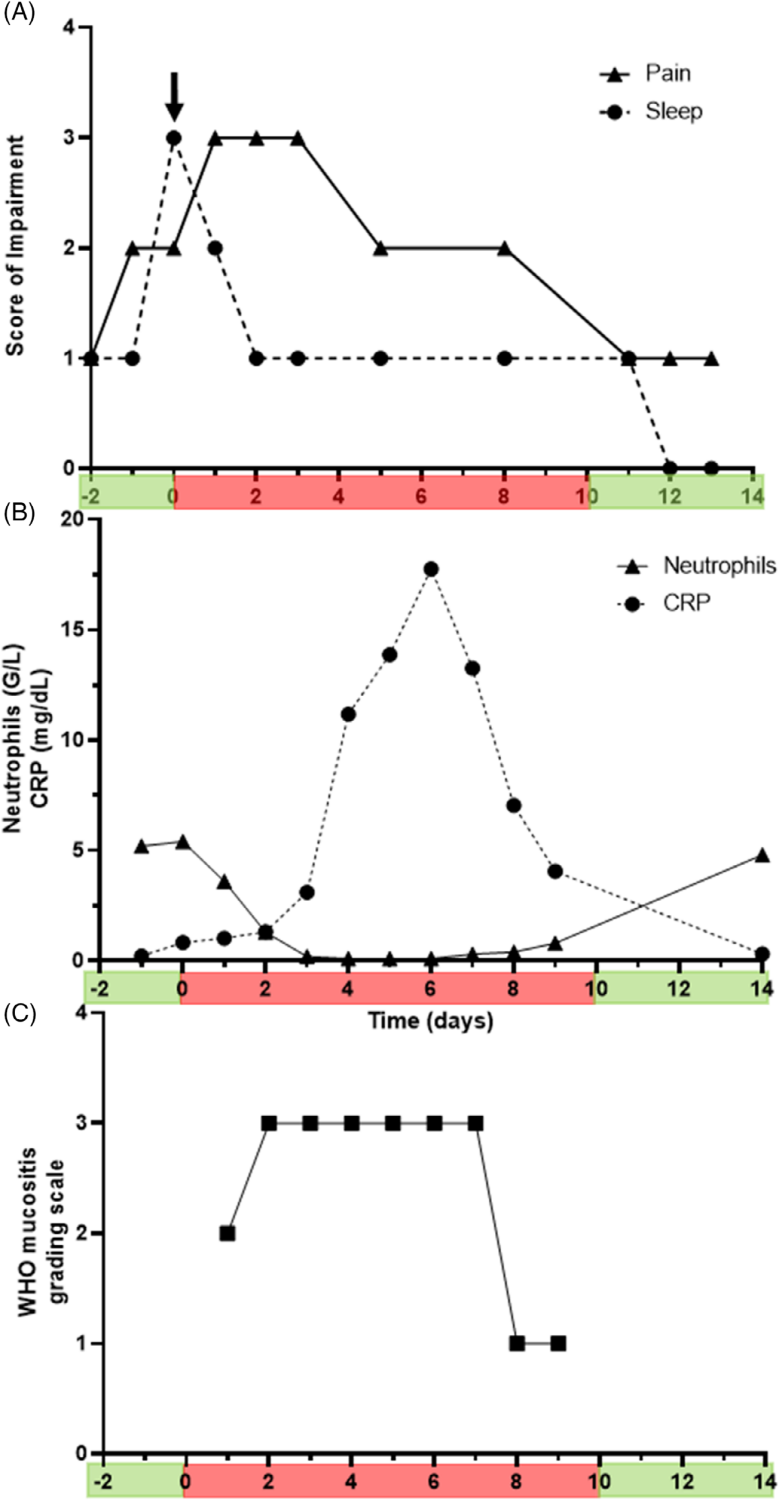
Medical intervention by the healthcare team at an outpatient therapy episode. Graphs display self‐assessment of pain and sleep (A), serum concentration of CRP and neutrophils (B) and mucositis grading by healthcare providers (C) over time. The green bars indicate home‐care, the red bars in‐patient treatment. The arrow indicates the time when the parents were contacted by phone by the medical team

A 10‐year‐old male patient was diagnosed with Burkitt leukemia at the Medical University of Innsbruck. After completing a second block of a four‐drug induction regimen, he was sent home for a 16‐day treatment break. Three days after discharge, severe impairment of sleep and moderate pain development were detected with the ePROtect, which triggered a notification to the medical team (Figure 1(A): arrow). Immediately, the medical team contacted the parents by phone, who confirmed the child's self‐report, and a visit to the hospital was recommended. A few hours later, the patient was admitted to the hospital and presented in a reduced general condition and showed a pale skin color. His vital signs were temperature 37.8°C, heart rate 132/min, respiratory rate 24/min and blood pressure 85/55 mm Hg. The oral cavity showed diffuse mucosal erythema with whitish membranes and a few necrotic and hemorrhagic lesions in the buccal mucosa and lower lip. All other physical exams were normal. Laboratory tests upon admission showed a neutrophil count of 5.0 G/L and a negative C‐reactive protein (CRP) (Figure 1(B)). Because herpes simplex mucositis was suspected, the patient was started on intravenous acyclovir (500 mg/m^2^/d), hydration was performed and intravenous analgesia (acetaminophen 15 mg/kg every 6 h) initiated. The following day, the patient reported improved sleep (Figure 1(A)), but he was unable to drink fluids or eat solid food (World Health Organization mucositis III, Figure 1(C)). On day 3 intravenous piritramide, patient‐controlled analgesia, was added for pain relief (Figure 1(A)). The next day, the patient deteriorated further, became febrile with progressive mucositis and showed severe neutropenia with increased CRP (4 mg/dL) (Figure 1). He was subsequently started on intravenous antibiotic treatment with meropenem (60 mg/kg/d), total parenteral nutrition was initiated and packed red blood cells and platelet transfusions were continued as needed. Self‐reported physical symptom burden slowly decreased over the following days (Figure 1(A)), despite maximal CRP increase to 17.1 mg/dL (Figure 1(B)). On hospitalization day 7, CRP levels started to decline, his mucositis began to resolve and his oral food and fluid intake improved steadily but slowly (Figure 1(B),(C)). From day 9 onwards, neutrophil count gradually recovered and analgesia and antibiotics were terminated. The patient was discharged on day 10 in good clinical condition with no self‐reported impairments (Figure 1(A)).

## 3 DISCUSSION

National healthcare systems are increasingly demanding the use of PROMs in daily healthcare in order to provide more responsive service delivery and to meet patients' rights to enhanced self‐determination.^7^ Studies from adult oncology have shown that the use of PROMs may not only lead to improved treatment quality and adherence, but also to longer overall survival.^8^, ^9^

The clinical reality in pediatric oncology is disappointing: a recent analysis of registered clinical trials showed that less than 8% of clinical studies used PROMs and less than 1% have used PROMs as primary endpoint.^10^ Since only few pediatric studies have made powerful insights into deterioration of quality of life under chemotherapy, it is unclear how these measures should be integrated into daily healthcare service delivery. So far, these studies have collected data over long time intervals (e.g., 1‐6 months) and there are even fewer reports on how digital technology and daily clinical care are integrated at the individual patient level.^11^, ^12^

Our case demonstrates that web‐based PROM monitoring enabling real‐time symptom monitoring by the healthcare providers has a number of potential benefits. Patients and their families feel in better hands if their needs are responded to promptly and before they become clinically evident. Moreover, the clinicians have a better tool that facilitates decision‐making on whether to discharge or readmit a patient for hospital care. But most importantly, the ability of the patient to express him‐ or herself better, together with the experience of receiving effective symptom relief, can positively reinforce improvements in quality of life and influence clinical outcome.

Recent policy interest and advances in information technology have made it possible for web‐based systems, such as the one used here, to potentially be used in everyday clinical practice. Expanded use of such systems could also assist clinicians in conducting clinical studies and meeting the needs our healthcare systems. The results of such studies across whole patient populations will be eagerly awaited.

## CONFLICT OF INTEREST

B. Holzner and GR hold IPRs of the CHES software tool.

## AUTHOR CONTRIBUTIONS

All authors had full access to the data in the study and take responsibility for the integrity of the data and the accuracy of the data analysis. Conceptualization, R.C., A.M., B.H.; Methodology, R.C., A.M., D.R., B.H., J.L. and G.R.; Investigation, R.C., A.M., A.H., B.H., and G.K.; Formal Analysis, A.M., J.L., and R.C.; Resources, R.C., A.M.; Writing ‐ Original Draft, R.C., A.M.; Writing ‐ Review & Editing, R.C., A.M., G.K., B.H., D.R., B.H., and J.L.; Visualization, A.M.; Supervision, R.C.; Funding Acquisition, R.C.

## ETHICS STATEMENT

ePROtect has been in use since May 1, 2020 within a prospective longitudinal, single‐armed feasibility study at the Division of Childhood Oncology, Medical University of Innsbruck. The Ethics Committee at the Medical University of Innsbruck approved this study (EC number: 1055/2020); written informed consent was obtained from the child and its parents.

## Data Availability

We confirm to the journal policy for data availability. The data that support the findings of this study are available from the corresponding author upon reasonable request.
